# The Effect of Community Health Information System on Health Care Services Utilization in Rural Ethiopia

**DOI:** 10.4314/ejhs.v33i1.3S

**Published:** 2023-04

**Authors:** Setegn Tigabu, Girmay Medhin, Mulusew G Jebena, Tegene Legese Dadi, Daniel Tadesse, Mekdes Demissie, Fasil Walelign Fentaye, Zelalem Tazu, Shegaw Mulu, Yibeltal Kiflie Alemayehu, Alula M Teklu

**Affiliations:** 1 MERQ Consultancy PLC, Addis Ababa, Ethiopia; 2 Addis Ababa University, Aklilu Lemma Institute of Pathobiology; 3 Jimma University, Department of Epidemiology, Faculty of Public Health; 4 Hawassa University College of Medicine and Health Science, School of Public Health1; 5 College of Health and Medical Sciences, Haramaya University, Ethiopia; 6 Centre for Innovative Drug Development and Therapeutic Studies for Africa (CDT-Africa), College of Health Science, Addis Ababa University; 7 Wollo University, School of Public Health; 8 Addis Ababa University, Department of Statistics; 9 Federal Minister of Health, Ethiopia; 10 Department of Health Policy and Management, Jimma University, Ethiopia; 11 Department of Global Community Health and Behavioral Sciences, School of Public Health and Tropical Medicine, Tulane University, New Orleans, USA

**Keywords:** Community health information system, maternal and child health services, service utilization, health posts, Ethiopia

## Abstract

**Background:**

In Ethiopia, the community health information system (CHIS) is implemented at the health post (hp) level with the aim of improving service delivery and use. We conducted a national level assessment of CHIS utilization and explored the associations of CHIS utilization with use of antenatal care (ANC), postnatal care (PNC), institutional delivery and child immunization in rural Ethiopia.

**Methods:**

We conducted a cross-sectional study measuring community-based health service use and HP based CHIS assessment from March to May 2019. Data were collected from 343 HPs and 2,864 women who delivered in the last five years, and multistage sampling was used to select the study subjects. We used descriptive statistics for CHIS implementation and service utilization and multilevel logistic regression to investigate the association of CHIS implementation with maternal and child health care services use.

**Results:**

Fifty five percent of the HPs were implementing CHIS. These HPs were using a paper-based household data collection tool called family folder (FF). Of the HPs, one third implemented lot quality assurance sampling (LQAS) based data quality check and 60.4% documented and followed execution of decisions. Overall, among the eligible women, 40% used ANC, close to 50% of currently married women used ANC services; 28% of women that fall in the high wealth index category used PNC within 48 hours after delivery; and 86.1% of women who had at least a high school education delivered at a health facility. Implementation of CHIS and family folder utilization and conducting LQAS based data quality check in the HPs were significantly associated with increased odds of ANC, delivery, and vaccination services use.

**Conclusion:**

We found that better implementation of CHIS was associated with better maternal and child health service use which implies that increasing utilization of CHIS at HPs will improve mother and child health service use.

## Introduction

Maternal and child health (MCH) continue to be largely overlooked aspects of the healthcare system, leading to major risks associated with pregnancy and childbirth (1). In 2003, recognizing the burden of infectious diseases and unmet need for maternal and child health, Ethiopia launched the health-extension program (HEP) in agrarian areas, and later, adapted and scaled the program for pastoral and urban areas in 2010 (2). However, MCH services use remains limited in Ethiopia. According to the 2019 mini demographic health survey report, the prevalence of PNC is only 11.8%. Better figures have been achieved for ANC4 and institutional delivery (16.1% and 15.4% respectively), but these still remain low (3).

To support health care delivery, Ethiopia introduced a nationally standardized comprehensive health management information system (HMIS) in 2007 and CHIS in 2010 (4). The CHIS is designed using a unified data collection tool called family folder (FF) (5). FF is a paper-based tool created for each household that includes information about household characteristics, family members, and the status of implementing HEP packages. It also has different health cards useful for recording health conditions and care delivery for each family member. HEWs are responsible for implementing the FF at the HP level. The CHIS also has different forms, including kebele-level data compilation forms, demographic profiles, environmental sanitation profiles, different tally sheets, responsibility of the HEWs, and reporting formats (5,6).

Though the Ethiopian government reported that there is high level of implementation of HMIS and CHIS in HPs, there have been a number of challenges (7). Some of the challenges faced in HMIS utilization are inadequate skills for gathering and analyzing data among health care staff at lower levels, poor data quality in the reports, fragmented and cumbersome implementation, and inconsistent data capture (5, 8). These challenges result in a compromised ability to make informed data-driven decisions (9,10). For example, incomplete data entry is a barrier to accurate monitoring of key indicators, weakening one's ability to identify gaps in service delivery and threatening the effectiveness of implementation of the national health extension program (11).

Building a sustainable and reliably implemented health information system has become one of the strategies to improve MCH services in Ethiopia. For example, in the food-insecure Ethiopian highlands, proper utilization of FFs was significantly associated with better service use (9, 11). In a cross-sectional comparative study of 177 kebeles, community-based data for decision-making (CBDDM) was associated with improvements in most maternal and new-born health care services use (12). However, this study did not comprehensively measure the status of CHIS utilization based on the composite score of several items. Other studies have found variable effects of using CHIS; one study found positive results (8) while others reported either negative or non-significant relationships (11,12,10). To our knowledge, the level of CHIS utilization and its association with health care service use have yet to be thoroughly explored at the national level.

In this study, we evaluated the implementation status of CHIS in rural parts of Ethiopia and explored the associations of levels of CHIS implementation with ANC, delivery, PNC, and child vaccination.

## Materials and Methods

**Study setting**: During data collection, Ethiopia had nine regional states and two city administrations. Regions are further subdivided into zones, including the second level of administration, districts (or *woreda*) comprising the third-level administrative structure, and the lowest government budget center. The woredas are further subdivided into “kebeles,” which are the lowest administrative structure of the government. Kebeles are subdivided into smallest non-administrative units called “Gotes.”

Health service delivery in the country follows a three-tier system: third-level health care, which is being provided by referral hospitals; secondary-level health care at zonal hospitals, and the middle and primary health care units (PHCU), which provide the lowest level of essential health care services. The PHCU includes a district hospital and health centers to serve about 25,000 people, with each health center having five satellite HPs. The HEP was designed to provide and promote preventative services with minimum essential curative services.

**Study design and period**: The current study is based on the analysis of data extracted from a national HEP assessment survey data base (13). The national HEP assessment used a community- and facility-based multi-stage cross-sectional survey design and conducted with the broader objective of assessing the status of the HEP in Ethiopia (14). The facility survey used a two-stage and the community survey used a three-stage sampling method. The detailed sampling procedures are described elsewhere (13). Sampling weight was calculated using the inverse of the sampling probability of study units, taking multistage sampling into account. The data collection was done from March to May 2019 (13).

**Study location and participants**: We included 177 health posts to assess the effect of CHIS on service use (ANC, institutional delivery, PNC, child vaccination) and 343 heath posts (HEWs providing services at health posts) for the component of the study that assessed the implementation status of CHIS.

**Participants**: We used a sample of the data from the HEP national survey data. We took 2,864 women who were pregnant in the last five years for ANC services outcomes; 2,217 women who delivered in the last five years for institutional delivery outcomes; 1,518 women who delivered in the last two years for PNC services; and 751 children between 12 to 23 months for immunization coverage (defined as full vaccination outcomes).

**Data collection tools, method, and quality assurance**: The HP assessment tool was used to guide the assessment of CHIS utilization at the HP level. Household-level tools were used to collect data about utilization of health services, such as ANC, PNC, institutional delivery, and immunization. The data collection tools and methods used are described elsewhere (13).

### Measurements

**Outcome- MCH care services utilization**: The maternal and new-born health care practices expected to be improved as a result of utilizing different CHIS domains were (a) the coverage of the fourth ANC visits to an HP/facility, (b) institutional delivery, (c) receiving postnatal care within 48 hours of childbirth, and (d) full vaccination coverage for children according to the national guidelines.

**Exposure variable- Four domains of the CHIS**: Based on the national guideline indicators, the level of CHIS utilization was measured based on 20 items, each coded “1” for yes and “0” for no. The 20 items were sub-grouped into four domains: (a) implementation and family folder utilization (five items), (b) utilization of standard tools and reporting formats consistently (eight items), (c) conducting LQAS data quality checks (one item), and (d) the existence of a performance review team and consistent follow up of execution of decisions (six items). Cronbach's alpha was used to check internal consistency of all the items included in each of the four CHIS domains, with results from 0.73 to 0.86. Each sub-domain was changed to a dichotomous variable.

**HP measures**: These included HEW education, availability of infrastructure, regular electricity, improved water sources, sanitation facilities, communication equipment, and availability of tracers. All variables were dichotomous and coded 1 for “yes available” and 0 for “no/not available.”

**Data analysis**: Frequencies and proportions were used to summarize characteristics of study participants and HPs. A multilevel logistic regression analysis was used to investigate the association of utilization of different domains of CHIS and MCH services use after adjusting for age, marital status, wealth, level of education of study participants, and characteristics of HPs (i.e., level of HEWs' education, availability of rooms, availability of tracer drugs, and availability of basic amenities). This model accounted for possible underestimation of standard errors of coefficients, which could lead to an overstatement of statistical significance. This model also controlled for correlations that might exist between observations within the same cluster (women within the same HPs tended to be more alike in health services utilization than women who were individually chosen at random from the population at large). A 95% confidence interval was computed around the estimates, and weighted results were reported. The analysis was done with Stata 16 software.

**Ethical considerations**: Ethical approval for the national HEP assessment survey was obtained from the ethical review committee of the Ethiopian Public Health Institute with protocol number *EPHI-IRB-151-2018*. Verbal consent was sought from all participants and documented by the interviewers. All the respondents were informed about their right not to participate in the study and to withdraw at any point during the data-collection process even if they initially consented to participate.

The authors of this paper got access to the data without any violation of confidentiality statement on the Ethics because of their direct involvement in the HEP assessment. This was one of several manuscripts included in the dissemination plan of the assessment.

## Results

**Characteristics of HPs**: Out of 343 HPs included in this study, 88.3% of the HPs were standalone facility while the remaining were functioning in rooms in a shared block, 54.9% had level-four HEWs. One third (34.1%) of the HPs had electricity from any source, and 87.6% had latrine facility. In general, none had all basic amenities, 11.3% had tracer drugs on the day of data collection, and 78.5% had three or more rooms.

**Utilization of CHIS in the HPs**: Fifty-five per cent of the HPs were implementing the CHIS, nearly two-thirds (63%) compiled basic kebele-level information, and 54.8% were implementing FFs. Of these, 96.3% arranged FFs based on “Gote” and household number. Fifty-five per cent of the HPs displayed performance information on their walls, 55.9% of HP performance review teams developed action plans with responsible bodies for the identified intervention areas, and 60.4% of the HPs documented and followed the execution of their decisions.

Overall, (a) 33.2% of the HPs implemented CHIS and were utilizing FFs during data collection, (b) 34.1% were utilizing LQAS-based data quality checks; (c) 10% had a performance review team who strictly reviewed plan versus achievement and followed the execution of decisions, and (d) 20.4% of the HPs consistently used standard data-collection templates and reporting formats (Table 1).

**Table 1:** Utilization of different components of CHIS in the HPs, May 2019

CHIS domains (N=343)	Percent*	CHIS items	Per cent (N)*
CHIS implementation and FF utilization	33.2	Implement CHIS	55.4 (343)
		Compile basic kebele-level information	63.0 (343)
		Use FFs	54.8 (343)
		Arrange FFs	96.3 (188)
		Folders updated	75.0 (188)
Utilization of standard formats and reporting system	20.4	Service delivery report	77.0 (343)
		Disease report	66.5 (343)
		Quarter report	59.8 (343)
		Annual report	50.7 (343)
		PHEM report	64.7 (343)
		Data collection tools	54.5 (343)
		Copy of reports	82.2 (343)
		Classification of diseases	52.1 (342)
LQAS	34.1	LQAS done	34.1 (343)
Performance review, follow-up, and execution of decisions	10	Display HEP performance information	55.0 (140)
		Has performance-monitoring team ever done performance review	83.6 (140)
		Availability of most recent performance review team meeting minutes	53.6 (112)
		Performance review team identified areas of interventions to improve performance	69.1 (110)
		Performance review team developed action plans with responsible bodies for the intervention areas identified	55.9 (111)
		HP documented and followed execution of decisions	60.4 (111)

* Weighted HEP: Health Extension Program

**Characteristics of women and health service use**: Overall, among the eligible women, 40% (n=2,864) used ANC, 28.9% (n=1,518) used PNC, and 53.0% (n=2,217) delivered in health facilities. Only 38.6% (n=751) of children between the ages of 12 and 23 months were fully vaccinated. Use was consistently lower in pastoral compared to agrarian communities, with the lowest rates in ANC4 and PNC. Higher rates of use for ANC were seen among women in agrarian (50.7%) compared to pastoral (20.7%) settings. More than one-fourth (28.3%) of women that fall in the high wealth index category used PNC within 48 hours after delivery, and 86.1% of women who had at least a high school education delivered at a health facility. Close to 50% of currently married women used ANC services. Nearly 42% of children aged 12 to 23 months whose mothers were 30–49 years of age had all full basic vaccinations (Table 2).

**Table 2:** Socio-demographic characteristics of study-participating women and health services utilization in the catchment of 177 HPs, May 2019, Ethiopia

Variables		ANC4 (N=2,864)		PNC (N=1,518)		Delivery (N=2,217)		Vaccination	

		Number	Percent*	Number	Percent*	Number	Percent*	Number	Percent*
Residence	Pastoral	218	20.7	96	17.7	203	30.3		
	Agrarian	929	50.7	343	25.6	966	57.5		
Wealth	Low	349	54.3	118	22.7	347	53.7	75	40.2
	Middle	213	55.6	88	21.4	222	54.2	44	33.8
	High	585	45.7	233	28.3	600	59.3	87	35.3
Formal Education	No education	658	44.0	245	21.1	670	52.0	121	35.9
	Primary	397	58.9	158	29.9	396	59.1	73	38.7
	Secondary and above	92	50.1	36	37.2	103	86.1	34	34.8
Marital Status	Currently Married	1075	49.6	410	25.0	1,102	56.7	197	36.7
	**Others	72	42.9	29	33.8	67	52.1	9	38.2
Age	<30	704	48.4	290	23.9	730	57.6	131	34.1
	30–59	443	50.6	149	27.7	439	54.7	75	41.5

Overall		1147	40.0	439	28.9	1,169	52.7	206	36.8

* Weighted

** Others includes: single, divorced, separated or widowed

**Figure 1:**
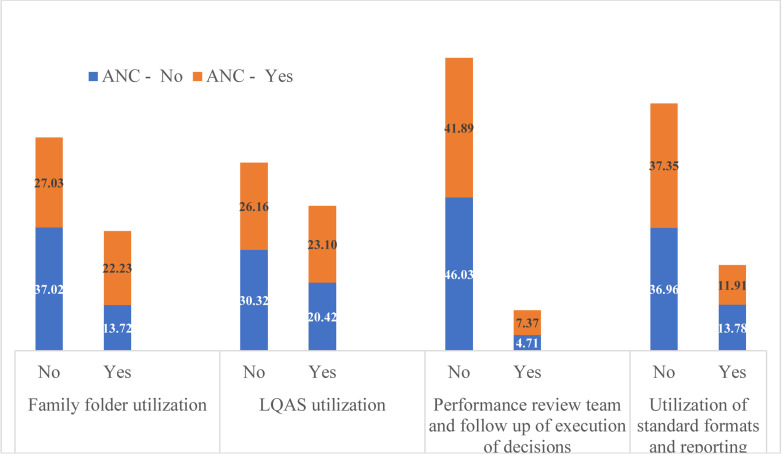
ANC services utilization of study participants by domains of CHIS utilization of HPs, May 2019, Ethiopia

**Figure 2:**
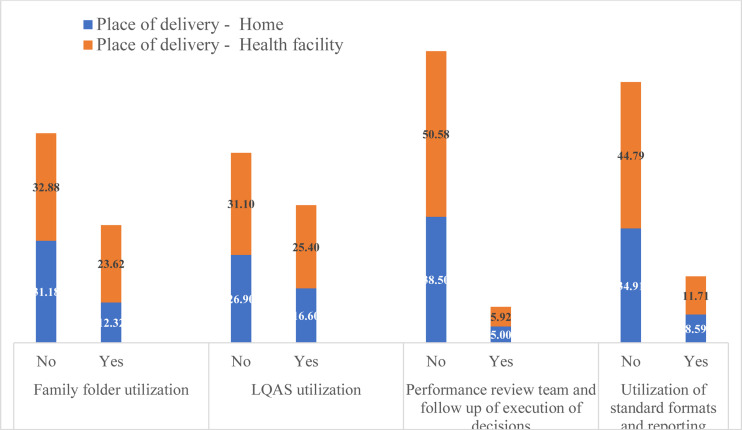
Institutional delivery services utilization of study participants by domains of CHIS utilization of HPs, May 2019, Ethiopia

**Figure 3:**
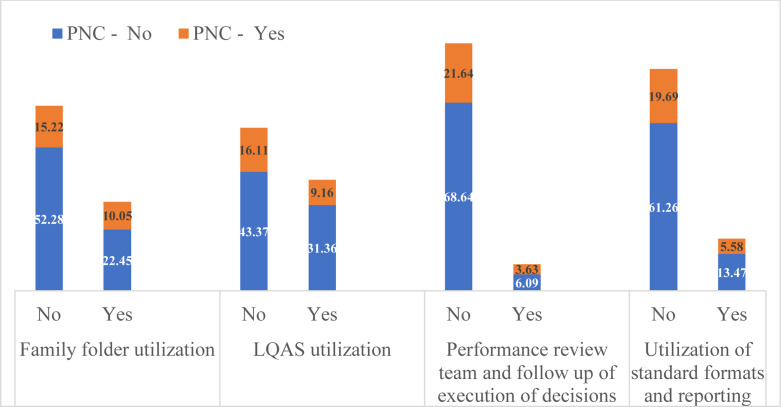
PNC services utilization of study participants by domains of CHIS utilization of HPs, May 2019, Ethiopia

**Figure 4:**
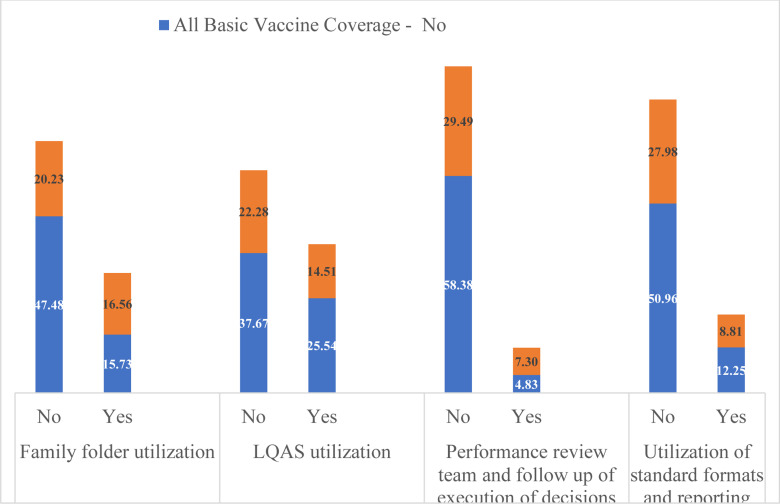
All basic vaccine coverage services utilization of study participants by domains of CHIS utilization of HPs, May 2019, Ethiopia

**MCH services by different CHIS domain use**: MCH services use was relatively high in the catchments of HPs where there were CHIS implementation, FF utilization, and implementation of LQAS-based data quality checks (figures 1 - 4). Roughly two-thirds (64%) of surveyed women were in the catchment of HPs that were not implementing CHIS and using FFs in the study period. ANC4 rates were lower for women served by these HPs (27.03%) compared to those (22.23%) served by CHIS-implementing and FF-using HPs (Figure 1). Similar results were seen regarding facility-based delivery, PNC, and vaccination services in CHIS-implementing and -non-implementing HPs (Figures 1 - 4).

After adjusting for potential confounding variables, implementation of CHIS and FF utilization, and LQAS-based data quality checks were significantly associated with increased odds of ANC, delivery, and vaccination services use. A woman in the catchment of HPs that implement CHIS and use FFs is two times more likely to use ANC services (AOR=2.05, 95% CI (1.37,3.07)) and 2.1 times more likely to deliver in health facilities (AOR=2.10, 95%CI (1.19,3.71)). A child 12–23 months of age whose mother lives in the catchment of a CHIS-implementing HP is 9.5 times more likely to be vaccinated (AOR=9.52, 95% CI (2.66, 34.14)). Similarly, a woman in the catchment of HPs that conduct LQAS-based data quality checks is 86% more likely to use ANC services than a woman in the catchment of HPs that do not conduct such checks.

These significant associations were also maintained after adjusting for wealth quintiles, educational level of the mother, marital status, maternal age, number of HEWs in the HP, number of HP rooms, and availability of tracer drugs (table 3). PNC services utilization of women in the catchment of HPs which were implementing CHIS was more or less similar to those women residing in the catchment of HPs which were not implementing CHIS (Table 3).

**Table 3:** Association of CHIS domains on MCH services use May 2019, Ethiopia

CHIS Utilization	Domains	ANC (N=2864)	Delivery (N=2217)	PNC (1518)	Immunization (N=751)

		OR (95% CI)	OR (95% CI)	OR (95% CI)	OR (95% CI)
Family folder utilization	Crude effect	2.09 (1.43, 3.05)	2.19 (1.28, 3.73)	1.30 (0.73, 2.32)	7.84 (2.23, 27.57)
	Adjusted effect*	2.05 (1.37, 3.07)	2.10 (1.19, 3.71)	1.25 (0.67, 2.34)	9.52 (2.66, 34.14)
Utilization of standard formats	Crude effect	1.08 (0.73, 1.20)	1.21 (0.70, 2.08)	0.94 (0.47, 1.88)	1.7 (0.44, 6.66)
	Adjusted effect*	1.08 (0.72, 1.63)	1.42 (0.75, 2.67)	0.97 (0.47, 1.98)	2.40 (0.56, 10.25)
LQAS-based data quality check	Crude effect	2.02 (1.46, 2.81)	2.81 (1.47, 2.45)	1.15 (0.65, 2.05)	3.09 (0.85, 11.17)
	Adjusted effect*	1.86 (1.29, 2.69)	1.30 (0.75, 2.25)	1.00 (0.54, 1.85)	3.61 (0.98, 13.35)
Follow up and execution of decisions	Crude effect	1.94 (1.13, 3.37)	0.70 (0.35, 1.40)	1.24 (0.55, 2.79)	3.40 (0.47, 24.49)
	Adjusted effect*	1.91 (1.09, 3.36)	0.65 (0.36, 1.19)	1.29 (0.56, 2.95)	3.61 (0.47, 27.54)

* Adjusted wealth, educational level, marital status, age category level and number of hews, number of available rooms, and availability of tracer drugs

## Discussion

In rural Ethiopia, implementation status of CHIS was very low, and its likelihood of proper implementation at HP was positively influenced if the HP staff composition includes level-four HEWs. However, nearly half of the HPs do not have this type of staff composition, and we did not find HPs that fulfill basic amenities. CHIS implementation, utilization of FFs, and conducting LQAS were positively associated with increased odds of MCH services use that include ANC, delivery, and vaccination services. CHIS implementation in rural Ethiopia was relatively lower than the level reported by the Ministry of Health using routinely collected program data (7). Two possible explanations for the observed difference could be data collection methods and potential bias towards wanting to report high performance by program implementers at a grassroots level. HPs that already implemented CHIS are more likely to arranging FFs based on “Gote” and household number than the overall implementation of CHIS in rural parts of the country. This finding aligns with the findings of previous smaller-scale studies (10,15,16). In the eastern part of Ethiopia, utilization of overall health information system was 53.1% (15). Overall health information system utilization was 45.8% in East Gojam and 32.9% in Jimma (10,16). A high level of routine health information system utilization among health professionals was also reported in the North Gondar Zone (17). These differences might be attributed to the difference in capacity and engagement of health professions involved in the implementation of CHIS.

More than half of the HPs displayed HEP performance information. Greater number of HPs documented and followed the execution of their decisions, which is in line with previous findings (15).

Implementation of CHIS and FF utilization were significantly associated with a high level of health service use. A woman in the catchment of HPs with CHIS implementation and FF utilization were two times more likely to deliver at a health facility compared to a woman in the catchment of HPs not utilizing CHIS. Similar association was observed in a study that was designed to test the effectiveness of CBDDM before and after implementation (12). Implementation of CHIS and FF utilization has even a relatively higher effect when it comes to immunization: A child aged 12–23 months is 9.5 times more likely to receive all full basic vaccinations if that child belongs to a catchment of HPs where there is CHIS implementation and utilization of FFs. This is likely due to the simplicity created by FFs in identifying a woman or household requiring health services from HEWs. HEWs are now better equipped to use their own data for decision-making, as they can now review monthly performance towards targets and monitor progress to improve prevention as well as clinical practices.

Conducting LQAS-based data quality checks in the HPs has significant association with increased health service use. LQAS improves maternal and child (household) data in source documents, allowing for improved client follow up and better management of beneficiaries. This study is a national-level assessment and tried to measure CHIS implementation and utilization based on different domains, which gives a complete picture of the utilization of information system at the source. However, because of the minimum number of eligible children in the selected households, which resulted in minimum sample size, the association between domains of CHIS and full basic vaccination were unstable.

In conclusion, the low level of CHIS implementation in rural areas observed in the current survey implies the need for strong monitoring and supportive supervision from regional and woreda-level supervisors and refresher training for hews that focuses on the usefulness of better implementation. Positive associations for proper utilization of different domains of CHIS with health services use implies that any investment aimed to improve CHIS utilization will have a strong health outcome return in terms of improving mother and child health, contributing towards long-term universal coverage. Hence, the low level of CHIS utilization in the current study warrants special attention.

## References

[1] Federal Ministry of Health, Regional Health Bureaux (2006). Ethiopia Health Sector Development Programme (HSDP II).

[2] Ethiopia Ministry of Health (2010). Health Sector Strategic Plan III.

[3] CSA (2014). Ethiopia Mini Demographic and Health Survey.

[4] Advancing Partners & Communities (2019). Health management information system scale-up project in Ethiopia: a five-year journey to better health information systems (Internet). Anuual Report.

[5] Consulting VW (2009). Health information systems in developing countries.

[6] Policy, Planning M and ED (2019). Agrarian community health information system implementation manual.

[7] Ministry of Health (2016). Ethiopia's experience on strengthening community health information systems, data quality and data use 16-18.

[8] Kare C, Tariq A (2013). Community health information system for family-centered health care: scale-up in southern nations, nationalities and people's region. Quarterly Health Bulletin of Ministry of Health of Ethiopia.

[9] Kitaw Y, Ye-Ebiyo Y, Said A, Desta H, Teklehaimano A (2007). Assessment of the training of the first intake of health extension workers. Ethiop J Heal Dev.

[10] Shiferaw AM, Zegeye DT, Assefa S, Yenit MK (2017). Routine health information system utilization and factors associated thereof among health workers at government health institutions in East Gojjam Zone, Northwest Ethiopia. BMC Med Inform Decis Mak.

[11] Hirvonen K, Berhane G, Woldu T (2020). Assessing community health information systems: evidence from child health records in food insecure areas of the Ethiopia n highlands. Matern Child Health J.

[12] Karim AM, Fesseha Zemichael N, Shigute T, Emaway Altaye D, Dagnew S, Solomon F (2018). Effects of a community-based data for decision-making intervention on maternal and newborn health care practices in Ethiopia : a dose-response study. BMC Pregnancy Childbirth.

[13] Alemayehu Yibeltal K, Teklu Alula M National assessment of the health extension program in Ethiopia: Study protocol and key outputs.

[14] Wang H, Tesfaye R, Ramana GN V, Chekagn CT (2016). Ethiopia health extension program (Internet).

[15] Teklegiorgis K (2014). Factors associated with low level of health information utilization in resources limited setting, eastern Ethiopia. Int J Intell Inf Syst.

[16] Ouedraogo M, Kurji J, Abebe L, Labonté R, Morankar S, Bedru KH (2019). A quality assessment of health management information system (HMIS) data for maternal and child health in Jimma Zone, Ethiopia. PLoS One.

[17] Dagnew E, Woreta SA, Shiferaw AM (2018). Routine health information utilization and associated factors among health care professionals working at public health institution in North Gondar, Northwest Ethiopia. BMC Health Service Research.

